# Tracking white-matter brain modifications in chronic non-bothersome acoustic trauma tinnitus

**DOI:** 10.1016/j.nicl.2021.102696

**Published:** 2021-05-08

**Authors:** Chloé Jaroszynski, Arnaud Attyé, Agnès Job, Chantal Delon-Martin

**Affiliations:** a Univ. Grenoble Alpes, Inserm U1216, Grenoble Institut Neurosciences, 38000 Grenoble, France; b Univ. Grenoble Alpes, CHU Grenoble Alpes, 38000 Grenoble, France; c Institut de Recherche Biomédicale des Armées, IRBA, Brétigny s/Orge, France

**Keywords:** AF, Arcuate Fascicle, AFD, Apparent Fiber Density, ATR, Anterior Thalamic Radiations, CA, Anterior Commissural bundle, CC6, Corpus Callosum, isthmus part, CG, Cingulum, CSD, Constrained Spherical Deconvolution, CST, Cortico-Spinal Tract, DTI, Diffusion Tensor Imaging, DWI, Diffusion Weighted Imaging, FA, Fractional Anisotropy, FOD, Fiber Orientation Distribution, also known as fODF, fiber Orientation Density Function, FPT, Fronto-Pontine Tract, FX, fornix, IFO, Inferior Fronto-Occipital fasciculus, ILF, Inferior Longitudinal Fasciculus, HCP, Human Connectome Project, HL, Hearing Loss, MNI, Montreal Neurological Institute, Acoustic trauma tinnitus, Diffusion imaging, Deep learning, Constrained spherical deconvolution, Tractography

## Abstract

Subjective tinnitus is a symptom characterized by the perception of sound with no external acoustic source, most often accompanied by co-morbidities. To date, the specific role of white matter abnormalities related to tinnitus reaches no consensus in the literature. The goal of this study was to explore the structural connectivity related to tinnitus percept per se, thus focusing on a specific population presenting chronic non-bothersome tinnitus of similar etiology (noise induced) without co-morbidities. We acquired diffusion-weighted images with high angular resolution in a homogeneous group of mildly impacted tinnitus participants (n = 19) and their matched controls (n = 19). We focused the study on two subsets of fiber bundles of interest: on one hand, we extracted the acoustic radiation and further included any intersecting fiber bundles; on the other hand, we explored the tracts related to the limbic system. We modeled the diffusion signal using constrained spherical deconvolution. We conducted a deep-learning based tractography segmentation and mapped Apparent Fiber Density (AFD) on the bundles of interest. C, as well as Fractional Anisotropy (FA) and FOD peak amplitude for comparison. Between group statistical comparison was performed along the 27 tracts of interest controlling for confounding hearing loss, tinnitus severity, and duration since onset. We tested a potential correlation with hearing loss, tinnitus duration and tinnitus handicap score along these tracts. In the tinnitus group, we observed increased AFD related to chronic tinnitus percept after acoustic trauma in two main white matter regions. First, in the right hemisphere, in the isthmus between inferior temporal and inferior frontal cortices, in the uncinate fasciculus (UF), and in the inferior fronto-occipital bundle (IFO). Second, in the left hemisphere, underneath the superior parietal region in the thalamo parietal tract and parieto-occipital pontine tract. Between-group differences in the acoustic radiations were not significant with AFD but were with FA. Furthermore, significant correlations with hearing loss were found in the left hemisphere in the inferior longitudinal fasciculus and in the fronto-pontine tract. No additional correlation was found with tinnitus duration nor with tinnitus handicap, as reflected by THI scores. The regions that displayed tinnitus related increased AFD also displayed increased FA. The isthmus of the UF and IFO in the right hemisphere appear to be involved with a number of neuropsychiatric and traumatic disorders confirming the involvement of the limbic system even in chronic non-bothersome tinnitus subjects, potentially suggesting a common pathway between these pathologies. White matter changes underneath the superior parietal cortex found here in tinnitus participants supports the implication of an auditory-somatosensory pathway in tinnitus perception.

## Highlights

• Tractography was compared between two groups of tinnitus and control participants.
• Diffusion was modeled with ss3t-CSD allowing apparent fiber density (AFD) calculation.
• 27 bundles of interest were chosen for their link to the auditory and limbic systems.
• AFD was significantly increased in the tinnitus group in the right frontal isthmus.
• AFD in the acoustic radiations was not significantly different between the groups.

## Graphical abstract

**Figure:**
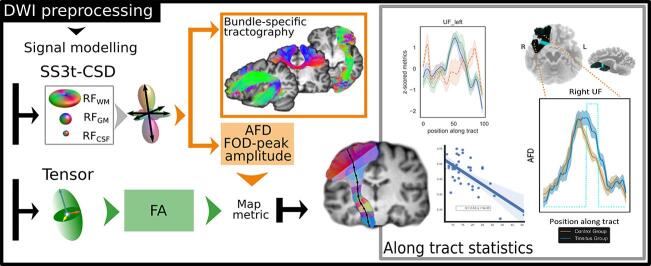

## 1 Introduction

Tinnitus consists in the conscious perception of sound in the absence of a corresponding source. It is an inherently complex condition to assess, since it cannot be characterized objectively (Jackson et al., 2019) and is symptomatic of a range of diseases (Baguley et al., 2013, Jastreboff, 1990, Møller, 2016). Its characterization mainly relies on the subject’s description of their condition and its possible etiology (Crummer and Hassan, 2004, Heller, 2003), which is why understanding the underlying mechanisms responsible for this symptom remains a hot topic in neuroscientific research.

In the case of tinnitus following acoustic trauma, defined as an injury to the inner ear often caused by exposure to high decibel noise, it has been suggested that the symptom may originate from cochlear deafferentation (Eggermont and Roberts, 2015, Henry, 2016, Jastreboff, 1990, Lanting et al., 2009). Impacting the auditory pathway up to the cortex and leading to neural plasticity, this model is the historical view on tinnitus. It has been empirically contradicted as unique explanation by cases of tinnitus subsisting for instance after acoustic nerve section (House and Brackmann, 1981). In contrast with this hypothesis, some authors suggest that tinnitus persistence could also involve systems different from the auditory pathway. Reviews of neuroimaging studies have investigated grey-matter or connectivity modifications in tinnitus subjects and underline the role of the limbic system in tinnitus perception, at the level of tinnitus generation and maintenance (Adjamian et al., 2014, Simonetti and Oiticica, 2015). Further studies have shown the implication of the limbic system (Chen et al., 2017, Leaver et al., 2016), as well as the default mode network (Elgoyhen et al., 2012), the right executive control network (Kandeepan et al., 2019), but also the autonomic pathway (Vanneste et al., 2013) or a proprioceptive pathway involving the middle-ear (Job et al., 2016). In the latter, a model of tinnitus induced by acoustic trauma based on auditory, autonomic, limbic and middle ear proprioceptive pathways is proposed.

Yet, no clear picture emerges from these numerous studies. Why? The discrepancies found throughout the literature likely relate to co-morbidities associated with tinnitus. Indeed, subjects included in most studies present various levels of hearing loss, distress, differences in etiology or differences in coping strategies to manage their symptom, to name a few. The lack of consensus due to these confounding factors has led some authors to advocate for more homogeneous groups of participants (Koops et al., 2019, Schmidt et al., 2018, Wineland et al., 2012). First, considering the chronicity of tinnitus helps categorize the percept. Tinnitus is considered chronic when it has been at a constant state for at least 6 months (Tunkel et al., 2014). Second, quantifying the subjective experience using a visual scale or a questionnaire makes it more observable and comparable. Moreover, the subjective characterization of tinnitus as bothersome seems to constitute a criterion for medical intervention. Tinnitus is considered bothersome when negatively affecting sleep, concentration, emotions, and social enjoyment (Bauer, 2018). For clinical studies, Bauer encourages clinicians to distinguish patients with bothersome tinnitus from patients with non-bothersome tinnitus. In the field of tinnitus research, in order to deepen the understanding of the neural correlates of tinnitus percept *per se*, targeting chronic tinnitus with a low impact on daily life as evaluated by the Tinnitus Handicap Inventory (THI) limits the occurrence of confounding factors related to the presence of co-morbidities. In addition, focusing on tinnitus of acoustic trauma origin further improves the homogeneity of the recruited subjects. Finally, remaining confounds such as degree of hearing loss or age that may still appear between tinnitus and control participants must also be accounted for using appropriate statistical methods.

That said, the literature provides evidence that long term mechanisms come into play in the presence of chronic acoustic trauma tinnitus perception. It is now established that the central nervous system has a remarkable capacity for plastic change, even into adulthood, resulting from changes in input stimuli (Gage, 2004). The literature on structural plasticity suggests that white matter modifications, as measured with Magnetic Resonance diffusion weighted imaging (DWI), are reinforced by disease duration as in multiple sclerosis (Deppe et al., 2016), by chronicity of traumatic brain injury (Håberg et al., 2015) and psychological factors (Melloni et al., 2019), among others. The persistent subjective perception of a chronic sound, as experienced by tinnitus subjects after an acoustic trauma event, could also lead to structural as well as functional plasticity. Few studies have investigated the structural modifications of white matter fiber bundles in tinnitus subjects using DWI. One of the first studies to do so reported the involvement of auditory or auditory-limbic connectivity (Seydell-Greenwald et al., 2014). This is consistent with anatomical evidence of afferent and efferent connections between the auditory and the limbic system in the healthy brain (LeDoux, 2007, Sah et al., 2003, Weinberger, 2004, Winer and Lee, 2007) However, it was also found that changes in white matter were rather related to cofactors such as hearing loss, age, depression levels or to tinnitus loudness (Adjamian et al., 2014, Gunbey et al., 2017, Luan et al., 2019, Ryu et al., 2016, Seydell-Greenwald et al., 2014, Yoo et al., 2016). The question of the role of the limbic system specifically in non-bothersome tinnitus remains open. Other reports addressed WM connectivity alterations with matched groups, or while regressing confounding factors, and using Diffusion Tensor model (Benson et al., 2014, Schmidt et al., 2018). Schmidt’s subgroup study showed no differences with the control condition, supporting the idea again that homogeneous groups are needed for white matter differences to be elicited. Benson’s matched hearing loss study of tinnitus provided evidence of Fractional Anisotropy (FA) increasing in the tinnitus group in the left thalamic, frontal and parietal white matter. In the present study, we aim to focus on the WM alterations related to the chronic tinnitus perception *per se* with a specific focus given to the auditory and to the limbic pathways, based on reviews of the literature and models of noise induced chronic tinnitus (Job et al., 2016, Kapolowicz and Thompson, 2020, Kraus and Canlon, 2012, Langguth et al., 2019, Leaver et al., 2016).

Finally, additional heterogeneity in DWI studies may pertain to methodological issues: rapid progress in the field of DWI inevitably makes results difficult to compare (Scott-Wittenborn et al., 2017). Indeed, DWI developments have come a long way since diffusion modeling by tensor as performed with diffusion tensor imaging (DTI) (Farquharson et al., 2013), a model that assumes a single fiber orientation per voxel. DTI prevailed in previous studies on tinnitus and may remain relevant in brain areas where simple fiber configurations exist but has proved to be particularly limited in regions of crossing fibers (Douaud et al., 2011, Pierpaoli et al., 2001, Wheeler-Kingshott and Cercignani, 2009), which is the case for most white matter voxels (Jeurissen et al., 2013). Both improvements in data acquisition (high angular diffusion directions, multiple b-values, among others) and in computational modeling of the signal using Orientation Distribution Function (ODF)-based models were developed to solve complex cases of crossing or kissing fibers (Tournier et al., 2011). In practice, for tractography related applications, spherical deconvolution and its constrained versions (CSD) using a spherical harmonics basis offers a good trade-off between clinical feasibility and the model’s ability to accurately represent the underlying fiber populations (for a review, see (Dell’Acqua and Tournier, 2019)). Recent additional contributions of deep-learning methods for distortion correction and for fiber bundle segmentation have taken diffusion imaging processing a step beyond (Schilling et al., 2019, Wasserthal et al., 2019). These methods benefit from the increasing availability of large, standardized, high-quality datasets, such as provided by the Human Connectome Project (HCP), improving the robustness of fiber bundles delineation for use in neural network-based methods.

The aim of the present study was to investigate whether or not there may exist white matter modifications related to the chronic experience of tinnitus in a homogeneous group of participants with non-bothersome tinnitus induced by acoustic trauma. In this study, we took advantage of the latest methodological developments to revisit structural WM plasticity related to tinnitus.

## 2 Materials and methods

The study was approved by the Local Ethics Committee CPP Sud-est V, Ref: 10-CRSS-05 MS 14-52, and conducted in accordance with the Declaration of Helsinki. Informed written consent was obtained from all participants.

### 2.1 Participants

In the chronic tinnitus group, nineteen male participants (mean age 42.5 ± 12 years old, range 24 to 64 years) were included. Inclusion criteria were tinnitus of acoustic trauma origin, tinnitus duration of at least 6 months, tinnitus considered as non-bothersome by participants. Subjects having had ear surgery, or with objective tinnitus, neurological lesions or diseases, psychiatric disorders, or with MRI related contraindications were excluded. Tinnitus participants full description is given in Table 1A. Their tinnitus characteristics were: mean duration 11.6 ± 7.6 years, median = 12 years, range 0.5–25 years; 68% of bilateral tinnitus, 16% right lateralized and 16% left-lateralized; loudness (mean 4.4, range 2–8) assessed on a visual analog scale from 0 to 10; medium high pitched sizzling (n = 1) and high pitched whistling (n = 18). Etiology included acoustic trauma related to military and leisure activities (rifle noise, loud music). 9 participants were professional military and 9 had other backgrounds. They were offered to participate in the study by medical unit physicians in military regiments and by the Ear Nose and Throat department of military hospitals. Since most tinnitus subjects having experienced acoustic trauma were male and in order to avoid an odd male/female ratio, we chose to include only male participants, thus avoiding a gender bias factor in our study. In the control group, the 19 age and gender-matched participants were recruited by word of mouth and through a call for volunteers. They were allowed to have experienced tinnitus occasionally in the past. There were no military among the controls because there was a risk that hearing problems may not be mentioned by the participants out of concern for an impact on their careers.

**Table 1:** Demographic data of the population investigated. A. Tinnitus participants description, including: laterality (B: bilateral ; L: Left-sided; R: Right-sided), duration of tinnitus since onset, THI: Tinnitus Handicap Inventory score, origin of tinnitus (W: Working environment; AR: Army Rifle; M: Music), HL: Hearing Loss in decibels. B. General characteristics for the entire study group.

A						
Subject	Age (years)	Laterality	Duration (years)	THI	Origin of AT	HL (dB)
T1	56	B	12	6	W	34
T3	43	B	25	8	AR	14
T4	38	B	15	26	AR	20
T5	59	B	25	22	AR	46
T6	28	B	10	8	M	8
T7	42	B	2	14	AR	18
T8	43	B	14	32	AR	18
T10	42	B	18	24	M & AR	25
T11	51	L	10	44	AR	31
T12	40	R	16	18	AR	10
T16	26	L	1	12	AR	25
T17	60	B	17	12	AR	23
T18	24	B	1	26	M	6
T19	25	B	16	4	M	12
T24	41	R	2	12	M	22
T28	49	R	0.5	12	AR	23
T30	57	B	10	6	W	29
T35	51	L	10	16	M	9
T37	33	B	15	6	AR	11

B			
		Control Participants (n = 19)	Tinnitus Participants (n = 19)
THI	Mean (std)	–	16.2 (10.5)
	Range	–	4–44
Hearing Loss (dB)	Mean (std)	13.2 (3.8)	20.2 (10.3)
	Range	7–-0	6–46
Age (years)	Mean (std)	42.5 (11.9)	42.5 (11.6)
	Range	24–64	24–60
Duration (years)	Mean (std)	–	11.6 (7.6)
	Range	–	0.5–25

Prior to MRI examinations, hearing levels were measured in all participants using tonal audiometry at 0.25, 0.5, 1, 2, 4, and 8 kHz (Audioscan fx, Essilor®) and electroacoustic calibration was performed in accordance with French AFNOR standard S3007. The audiometer was equipped with Beyer dynamics DT48 earphones. Hearing thresholds were established using the automatic Hughson-Westlake procedure and hearing loss was expressed in dB HL. As described in (Job et al., 2020), hearing thresholds for frequencies of 1 to 8 kHz were significantly different between the groups (Mann-Whitney test, p < 0.05), with increased hearing loss above 4 kHz in the tinnitus group, consistent with acoustic trauma sequelae (Fig. 2).

Tinnitus participants were asked to complete a form collecting general information about their tinnitus, and the Tinnitus Handicap Inventory (THI) questionnaire to assess the intrusiveness of the symptom on daily life (Newman et al., 1996). The THI was translated to French and yielded a score out of 100 reflecting tinnitus severity. THI scored slight to mild in all participants (mean 16.2 ± 10.5, range 4–44) except for one participant with moderate tinnitus scoring THI = 44, reflecting the effort made in this study to constitute a homogeneous group of mildly impacted subjects.

Demographic data from the two groups of participants are reported in Table 1B. Noteworthy is the fact that tinnitus appears immediately after acoustic trauma, and therefore tinnitus duration is equal to the duration since the acoustic trauma event.

Autonomic status was assessed at rest in all participants by measuring heart rate variability before MRI acquisition using the signal from an ear photo-plethysmography (emWavePro, HeartMath ®) sensor. The signal was registered at 100 Hz sampling frequency and the peaks of the hemodynamic pulse were detected. The estimation of heart rate variability from these pulses are excellent at rest (Schäfer and Vagedes, 2013) allowing the analysis with Kubios software (https://www.kubios.com). Inter-peak intervals were further examined in time-domain, frequency-domain, and in nonlinear frameworks. Differences in autonomic status between the groups were tested using non-parametric Mann-Whitney U-tests in SPSS (IBM Corp. Released 2013). IBM SPSS Statistics for Windows, Version 22.0. Armonk, NY: IBM Corp®). No significant differences were found between the groups in the autonomic status during the course of the experiment.

### 2.2 MRI acquisitions

A 3 T Philips Achieva-TX system (Best, The Netherlands) from the IRMaGe MRI facility (Grenoble, France) with a 32-channel head coil was used for MRI scanning. The protocol included an anatomical T1-weighted sequence and a 15 min-long high angular resolution diffusion-weighted sequence.

The spin-echo diffusion-weighted sequence included 70 axial slices of 2 mm thickness with in-plane spatial resolution of 1.67 mm^2^, and a SENSE factor 2 was applied in the antero-posterior direction. 60 diffusion images were acquired with different gradient orientations at b = 1000 s/mm^2^ as well as one image at b = 0 s/mm^2^. The repetition and echo times (TR/TE) were set to minimum with actual values 5500/72 ms. The total sequence duration was 15:08 min.

The 3D-T1 MPRAGE (Magnetization Prepared Rapid Acquisition Gradient Echo) anatomical acquisition was the same for all participants, with following parameters: 150 axial slices, voxel size = 0.9*0.9*1.2 mm^3^, TI = 800 ms, TR = 25 ms, TE = 3.9 ms, flip angle = 15° and acceleration factor = 2.2.

### 2.3 Preprocessing of diffusion images

After conversion from raw DWI Philips data (PAR-REC format) to nifti, we performed denoising (Veraart et al., 2016) and removal of Gibbs artifacts (Andersson and Sotiropoulos, 2016) using corresponding functions from the MRtrix3 package (Tournier et al., 2019), version 3.0 RC3. In order to correct susceptibility-induced EPI distortions, we used an approach based on a synthetic non-diffusion image derived from the T1 using deep learning (Schilling et al., 2019). The synthetic b = 0 image was subsequently used to correct the DWI volumes for eddy current distortions and susceptibility-induced EPI distortions using FSL’s TOPUP and EDDY functions (Andersson et al., 2003, Graham et al., 2017). We ran a bias field correction with ANTs (Tustison et al., 2010), upsampled the images to a 1.3 mm isotropic resolution and proceeded with global intensity normalization (Raffelt et al., 2012). Finally, the individual DWI maps were rigidly registered to MNI space as advised for TractSeg use (Jenkinson et al., 2002, Wasserthal et al., 2019).

For each subject, the response to the diffusion phenomenon of single fiber white matter (WM), grey matter (GM) and CSF were obtained using an unsupervised method (Dhollander et al., 2019). These subject-specific responses were averaged across the group to obtain, for each tissue compartment, a unique group-specific response function. This ensures downstream comparability of the fiber orientation distribution (FOD) images and further computed metrics (Dell’Acqua et al., 2013, Raffelt et al., 2012).

We then modeled the diffusion signal voxel-wise using a single shell, three tissue type constrained spherical deconvolution (CSD) framework and estimated the FOD images as well as CSF and GM compartments with MRtrix3Tissue (https://3Tissue.github.io), a fork of MRtrix3 (Tournier et al., 2019). The peaks, or three largest maxima of the FOD expressed in a spherical harmonics’ basis, were extracted and used as input for bundle-specific segmentation and tractography.

### 2.4 Fiber bundles of interest (FOIs)

The scope of the study was narrowed down according to a tinnitus related hypothesis. We focused on the acoustic radiations (AR), the tracts that were found to intersect with them, and those documented as part of the limbic system atlas (Mori et al., 2009). These criteria thus targeted the following 33 FOIs. Those from commissural bundles: the isthmus of the corpus callosum (CC6) and the anterior commissural bundle (CA). Those from association bundles: fornix (FX), bilateral cingulum (CG), inferior fronto-occipital fasciculus (IFO), uncinate fasciculus (UF), inferior longitudinal fasciculus (ILF) and arcuate fascicle (AF). Those from projection bundles: acoustic radiation (AR), anterior thalamic radiations (ATR), superior thalamic radiations (STR), optic radiations (OR), fronto-pontine tract (FPT), parieto-occipital pontine (POPT), thalamo-parietal (T_PAR), thalamo-occipital bundle (T_OCC), striato-parietal (ST_PAR) and striato-occipital (ST_OCC).

### 2.5 FOI tractography

The bundle specific tractography pipeline TractSeg that allows to compute the individual tractography based on deep neural networks was used (Wasserthal et al., 2019). Briefly, it runs as follows: the subject-specific peaks image is fed to a deep neural network (U-NET) that was trained on a dataset of 105 subjects from the Human Connectome Project (HCP). The pretrained UNET returns three output types per subject. First, the mask of the FOI. Then, the ROIs corresponding to the beginning and end regions of each FOI. Finally, it returns the vector field representing the local fiber orientations, also known as tract orientation maps (Wasserthal et al., 2018). This in turn supports the probabilistic tracking of 10,000 fiber trajectories in each FOI and each subject.

Note that the acoustic radiations require a specific pipeline. As reported in the literature (Maffei et al., 2019), the acoustic radiations are notoriously difficult to segment and tract. Nonetheless, this was made possible using a different initial segmentation with Xtract (Warrington et al., 2020), which TractSeg was also trained upon. This was followed by a probabilistic tracking of 10,000 fiber trajectories in MRtrix3 using the segmentation results provided by Xtract.

As a quality control, a visual inspection of each FOI was performed for each subject. As the fornix and anterior commissure were not robustly reconstructed across all subjects, as expected with clinical data quality (Wasserthal et al., 2019), they were removed from further analysis.

### 2.6 Along FOI profiles of CSD and tensor derived metrics

In each bundle and each subject, the FOI profile was represented by the centroid fiber, estimated as the fiber minimizing the average distance to all fibers in the bundle (Garyfallidis et al., 2012, Yeatman et al., 2012). All fibers were divided into a fixed number of 100 segments. Fiber segments were then assigned the segment index of the closest centroid segment in an approach based on Bundle Analytics (Chandio et al., 2020). The metric values were evaluated along each segment of each streamline, and the average of these values was assigned to the corresponding centroid streamline segment.

The main metric used in this study was the Apparent Fiber Density (AFD), computed as the integral of the FOD lobe using afdconnectivity in MRtrix3 to access the results of FOD segmentation (Smith et al., 2020, Smith et al., 2013). As a complementary metric, we also used the FOD peak amplitudes and the tensor-derived fractional anisotropy (FA) for further comparison with previous literature.

Note that for ST-PAR and ST-OCC, the geometry of the bundles prevented this approach to find a clear segment assignment. These tracts were thus not included in the statistical analyses.

**Fig. 1:**
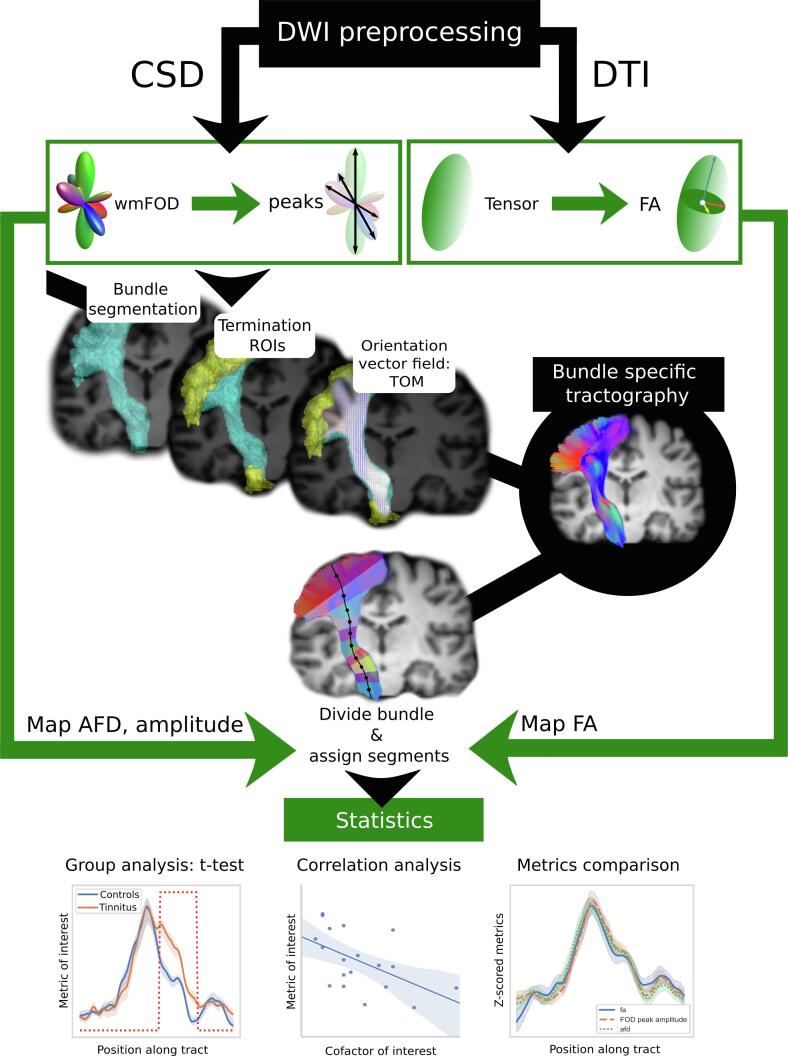
Pipeline of the processing used.

For sake of clarity, the overall pipeline is represented in Fig. 1

**Fig. 2:**
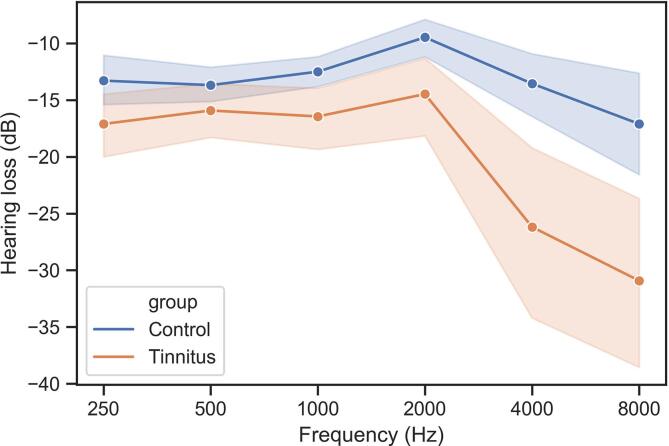
Frequency dependant levels of hearing loss in decibels in tinnitus and control participants.

### 2.7 Statistical analysis

#### 2.7.1 Between groups comparison

Age and hearing loss were reported to impact WM tractography (Yoo et al., 2016). Since both groups were matched in age, the co-factors that were retained in the analysis were the hearing loss, the THI and the tinnitus duration. In order to highlight any group differences related solely to tinnitus perception, we first linearly regressed all three confounds. Then we used a standard independent 2-sample *t*-test to compare metrics between tinnitus participants and controls, for each bundle and each position along the tract, using the additional statistical tools provided in TractSeg. Multiple comparison correction was performed (in TractSeg’s tractometry scripts) using non-parametric permutation testing with 5000 permutations (Nichols and Holmes, 2002), for multiple bundles and multiple positions along tracts correction. The permutation-based correction algorithm returns a family wise error (FWE) corrected value (p_FWE_ < 0.05), which provides the threshold below which the pvalue is considered significant. In addition, we considered a five neighboring segments cluster extent threshold for the discussion. For completeness, we provide the results obtained with 2 neighboring segments in the Supplementary Materials. The statistical threshold was again divided by three (additional Bonferroni correction for 3 metrics).

Results were plotted as tract profiles (Yeatman at al., 2012) displaying the evolution of the considered metric of interest for each group (mean and standard deviation) versus the position along the bundle. Overlaid are the statistically significant regions.

In order to check for any remaining influence of tinnitus laterality, we also performed the aforementioned analysis on the subgroup of bilateral tinnitus participants (n = 13).

#### 2.7.2 Correlation analysis

We investigated the potential influence of each cofactor on the metrics of interest using a correlation analysis. We estimated a linear regression model and computed the correlation between the cofactor of interest and the target metric, while subtracting the terms corresponding to the other confounds weighted by their respective regression coefficients. For hearing loss, we computed Pearson’s correlation with the metric values of the entire group, while for THI and tinnitus duration we used only the values from the tinnitus group. Permutation based correction for multiple comparisons was applied as described above.

Finally, we calculated the effect sizes (Cohen’s d for group differences and f2 for correlation analyses) and evaluated *post hoc* statistical power of significant results.

## 3 Results

### 3.1 Between group differences along tracts

**Fig. 3:**
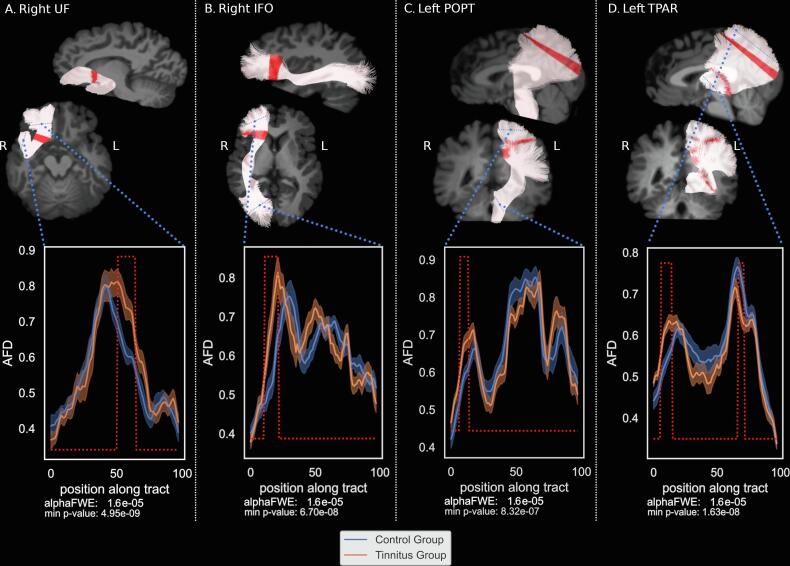
Statistically significant Apparent Fiber Density group differences.

The results with corresponding p-values and calculated power are summarized in Table 2. The two-sample Student’s *t*-test between both groups for all profiles revealed an AFD increase in the tinnitus group compared to controls in four FOIs: in the right uncinate fasciculus, the right inferior fronto-occipital bundle, the left thalamo-parietal bundle and in the left parieto-occipital pontine tract (Fig. 3 and Table 2A), after linear regression of all 3 cofactors. More specifically, in terms of locations along the FOIs, in the right UF (Fig. 3A), the significant area corresponds to the inferior part of the isthmus between the ventromedial prefrontal cortex and inferior temporal lobe. For the right IFO (Fig. 3B) the significant area was situated between the anterior insula and the ventromedial prefrontal lobe, in the superior part of the isthmus between frontal and temporal lobe. The left POPT (Fig. 3C) presented a significant increase in the fanning portion of the tract, close to the parietal cortex. For the left T-PAR (Fig. 3D), the significant increase in AFD in the tinnitus group is located in the fanning part of the parietal subdivision of the corona radiata close to the left POPT, while a significant decrease was observed in the region close to the thalamic seed (Fig. 3D).

**Table 2:** Between group differences along the tracts: (A) significant differences in AFD ; largest differences along the acoustic radiations; (B) significant differences in FOD-peak-amplitudes; (C) significant differences in FA. * values uncorrected for multiple comparisons.

Bundle names	Min p-value*	Segment number	T-value	Cohen’s d	power
(A)					
AFD					
IFO_right	6.70E−08	17	−6.764	2.255	0.980
POPT_left	8.32E−07	10	−5.942	1.981	0.907
UF_right	4.95E−09	62	−7.632	2.544	0.998
T_PAR_left	1.63E−08	11	−7.233	2.411	0.993
AR_right	4.70E−05	29	4.623	1.541	0.554
AR_left	0.00037	22	−3.930	1.310	0.312

(B)					
FOD-peak-amplitudes					
ATR_right	4.48E−06	14	5.394	1.798	0.802
IFO_right	1.6E−09	17	−8.017	2.672	0.999
UF_right	8.02E−08	57	−6.705	2.235	0.979
T_PAR_left	2.23E−07	9	−6.371	2.124	0.958

(C)					
FA					
AR_right	1.30E−05	34	5.037	1.679	0.702
IFO_right	1.64E−08	17	−7.231	2.410	0.994
OR_left	3.10E−08	51	7.018	2.339	0.990
POPT_left	2.22E−06	10	−5.622	1.874	0.855
STR_left	9.53E−06	13	−5.149	1.716	0.736
UF_right	1.86E−06	62	−5.680	1.893	0.867
T_PAR_left	6.50E−08	9	−6.775	2.258	0.982
T_OCC_left	2.44E−08	50	7.099	2.366	0.991

When considering specifically the acoustic radiations, no statistically significant differences in AFD were found between both groups. Nonetheless, at a weaker threshold (p_uncorrected_ < 0.0001), a difference could be elicited in the right acoustic radiation that we report for future studies at a location that bends just above the posterior part of the ventricle (Table 2A; Supp. Mat. S1). The low power found for this result does not robustly argue in favor of the involvement of the acoustic radiation in this subjective phantom percept. Note that this result holds for the segment between the medial geniculate body and the temporal lobe but not between the inferior colliculus and the medial geniculate since the acoustic radiation pipeline used here doesn’t allow the reconstruction in this portion of the tract.

Considering the FOD peak amplitudes metrics, differences between both groups were found significant in four bundles with increased FOD peak amplitudes for the tinnitus group in the right IFO, the right UF and left T-PAR (Supp. Mat. S3) in the same locations as with AFD and with decreased FOD peak amplitudes in the right ATR and another part in the left T-PAR (Table 2B). No significant difference was found in left POPT, the region found with AFD.

**Fig. 4:**
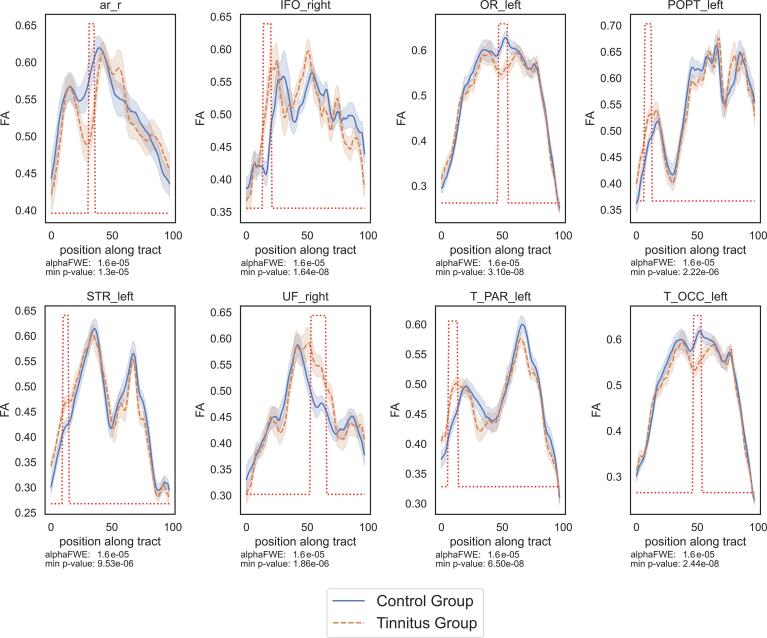
Significant results in the Fractional Anisotropy profiles.

Finally, for sake of comparison with the previous literature, the results with the FA metric (Fig. 4 and Table 2C) presented increased values in the tinnitus group as compared to controls in the right UF, the right IFO, the left T_PAR, the left POPT and the left STR . The locations in these bundles (except left STR) are the same as those with the AFD. The left STR location was also found with the AFD but with a smaller extent threshold of two neighboring segments. The tinnitus participants also present decreased FA values in the right AR, the left OR and the left T_OCC bundles (see statistical values in Supp. Mat. Table 3). This pattern of decrease was not found with the AFD analysis. Conversely, the region where decreased AFD was found in the left T-PAR also presents a similar profile in the FA analysis but does not reach significance.

We also present the results obtained for group comparisons with a smaller segment extent (n ≥ 2) for AFD (Supp. Mat. S1). Significant differences were then found in 12 additional fiber bundles: in the bilateral AF, the bilateral ATR, the right size of the isthmus of the CC (CC6), the left and right CG, the left FPT, the left part of the IFO, the right OR, the left UF and the left T_OCC. In these smaller segments, the Cohen’s d were higher than 1.8 with power above 0.80 (see statistical values in Supp. Mat. Table 1).

Additionally, the analysis of AFD in the subgroup of bilateral tinnitus participants reproduced the results found with the whole group analysis. Following results were elicited: a decrease of AFD for tinnitus participants in the middle of the right AF, an increase of AFD in CC6, in the middle of the right IFO, at the beginning of the right ILF, in the left OR, in the left STR, and in the right T-PAR, in the mirror region of T-PAR left found for the whole group analysis. Two more results were found: in the right AR, at the cortical extremity (joining Heschl’s gyrus), and in the left AR in the vicinity of the medial geniculate body, mirroring the result displayed in Supplementary Fig. S2.

### 3.2 Effect of hearing loss, THI and tinnitus duration

As expected, age and hearing loss were found to be significantly correlated, (r = 0.545, p = 0.0005) (Cardin, 2016, Yoo et al., 2016). No other significant correlation was found among cofactors.

**Fig. 5:**
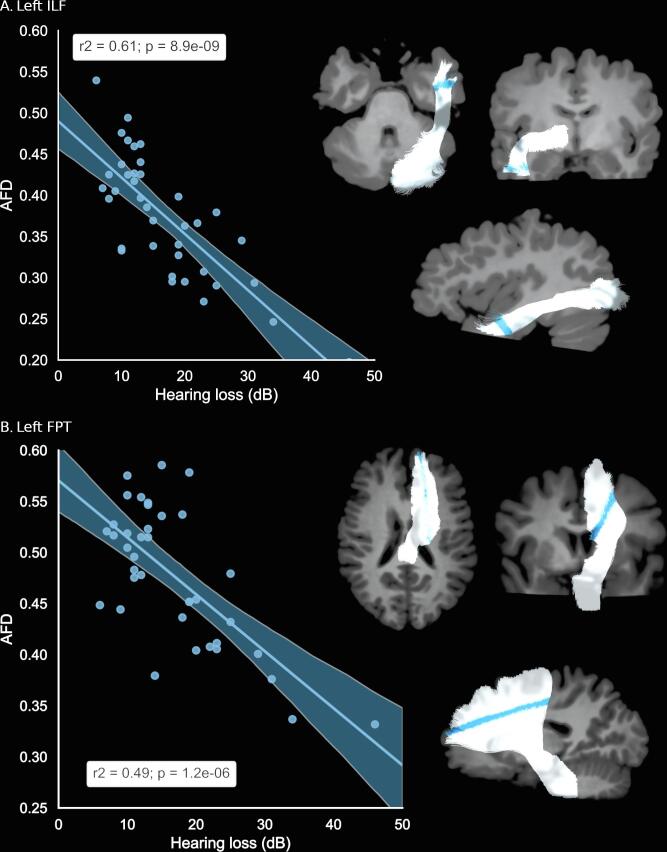
Significant correlation between AFD values and Hearing Loss.

The correlation analysis between AFD along the tracts and the three cofactors (hearing loss, tinnitus duration since onset and THI scores), returned significant results only for hearing loss. A negative correlation was found in the left FPT and in the left ILF (Fig. 5). The more severe the hearing loss, the lower the AFD in these tracts. In the left ILF, the correlation with HL was found significant in the inferior lateral part of the temporal lobe (Fig. 5A), while in the left FPT, the correlation was found significant in the fanning part of the dorsal frontal lobe (Fig. 5B). For sake of completeness and further studies on tinnitus, we report the tendency results (p_FWE_ < 0.1): at this threshold, the right UF in the inferior part of the temporal lobe presents an additional negative correlation with hearing loss (Supp Mat S4). No tendency for a correlation with THI or with tinnitus duration was found.

The FOD peak amplitudes correlation analysis rendered a single correlation found with hearing loss in the left FPT, similar to the result found in the left FPT for AFD. (Supp Mat Fig. S5)

**Fig. 6:**
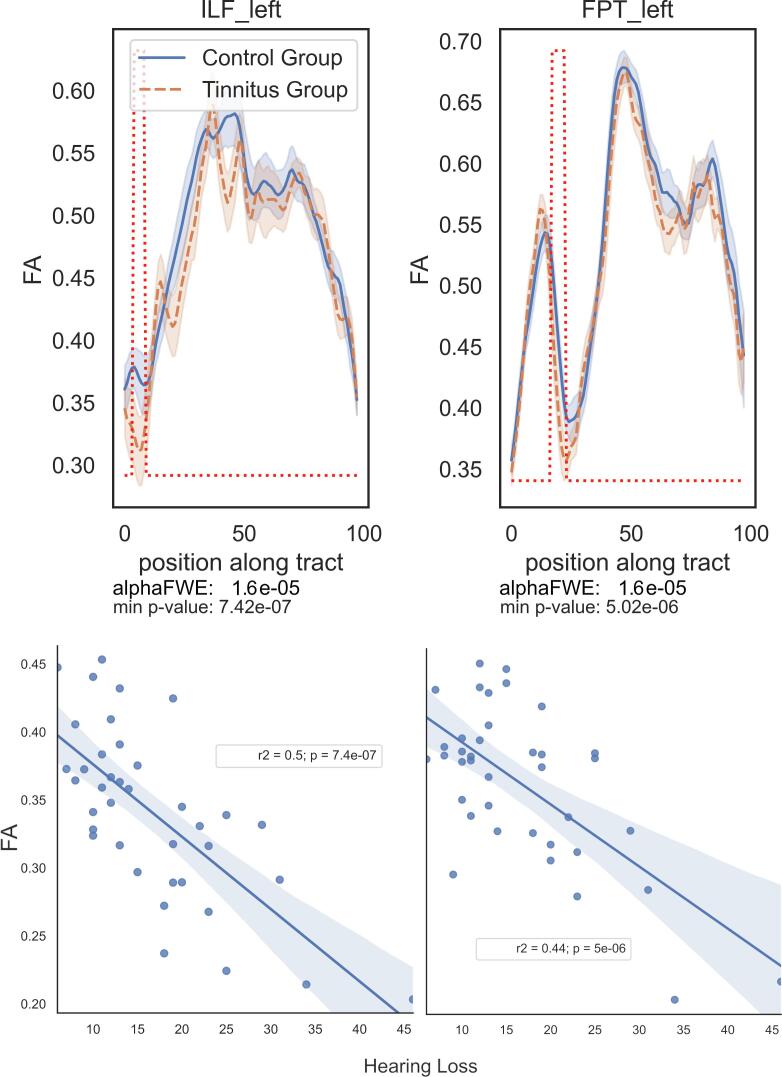
Profiles of FA and significant correlation with hearing loss in two bundles.

Finally, the correlation analysis with FA returned a negative correlation with HL in two tracts: the left FPT and the left ILF (Fig. 6), both in the same locations as with the AFD analysis. No significant correlation with THI nor with tinnitus duration was observed.

### 3.3 Metrics comparisons along FOIs

**Fig. 7:**
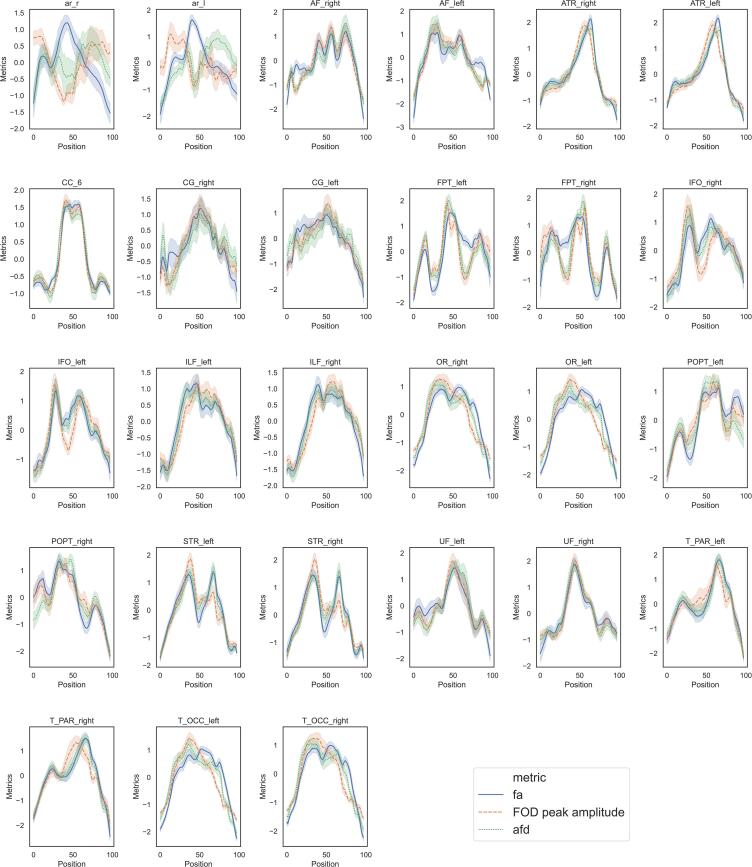
Profiles of the three metrics investigated in the study along the 27 tracts of interest.

The previous literature mainly reports FA metric based on the tensor model that assumes a single main fiber per voxel, while in this study we derived AFD and FOD peak amplitude metrics derived from a model accepting multiple fiber directions per voxel. It appears thus interesting to document these different metrics along the FOIs, for further comparison with previous DTI studies. We thus displayed the profiles of AFD, peaks amplitude and FA metrics taken in the control group (Fig. 7) along the FOIs after Z-transforming within each bundle for comparison. We observed strong similarities between AFD, FOD peak amplitudes, and FA profiles in most tracts. Noticeable is the difference in the acoustic radiations in their middle parts with lower AFD than FA, located approximately in region ld1 of the right insula. Other minor differences are located at the extremities of tracts (AF, CG, POPT, UF) or in their middle parts (CC6, FPT, OR, T_OCC). Note that right AR presents a particular profile with a decrease in its center (see Supp. Mat. S3), that might come from the particular geometry of this bundle.

## 4 Discussion

### 4.1 Summary/overview

The present study mapped apparent fiber density (AFD) profiles along fiber bundles of interest in chronic non bothersome tinnitus participants and controls. Fiber density is a metric of connectivity, reflecting the capacity of a fiber bundle to transfer information (Raffelt et al., 2017). While decreased AFD could thus reflect white matter atrophy, increased AFD could reflect white matter plasticity mechanisms, as typically supporting learning or adaptation to new sensory input (Sampaio-Baptista and Johansen-Berg, 2017, Stampanoni Bassi et al., 2019). White matter maladaptive plasticity is an erroneous modification of brain circuitry causing unwanted side effects, such as phantom perceptions. Evidence of cortical plasticity occurring after noise trauma both in the auditory pathway (Langguth et al., 2019) and in the limbic structures (Kapolowicz and Thompson, 2020) support the hypothesis of a joint contribution of both pathways for tinnitus to be perceived.

Here, we observed increased AFD related to chronic tinnitus percept after acoustic trauma in two main WM regions. First, in the right hemisphere, in the isthmus between inferior temporal and inferior frontal cortex, in its inferior part, in the UF, and in its superior part, in the IFO. Secondly, in the left hemisphere, in the superior parietal region of fiber bundles T-PAR and POPT. No significant differences were found in the acoustic radiations for AFD. Furthermore, significant correlations with hearing loss were found in the left hemisphere in the ILF and FPT. No additional correlation was found with tinnitus duration or with tinnitus handicap reflected by THI scores. The regions that displayed tinnitus related increased AFD also displayed increased FA.

Considering that the tinnitus group was mildly impacted by the symptom, that they had similar etiologies for that symptom and that confounding factors and multiple comparisons were taken into account in the statistical analysis, we rest assured that these results relate to the perception of tinnitus *per se* and are not contaminated by co-morbidities.

### 4.2 Involvement of the isthmus between inferior temporal and frontal lobes

The major result of this study emerged in the isthmus between inferior temporal lobe and inferior frontal cortex of the right hemisphere within two fiber bundles, the uncinate fasciculus and the IFO.

So far, the function of the uncinate fasciculus remains poorly known. It was recently described, with a virtual dissection study of a cohort of 60 participants (Hau et al., 2017) and using diffusion data of the Human Connectome Project (Briggs et al., 2018). It has been proposed to play a role in the limbic system, particularly in emotional regulation (Von Der Heide et al., 2013). This is of particular interest considering the involvement of limbic tracts that have been reported in tinnitus participants (Seydell-Greenwald et al., 2014). Indeed, in Seydell-Greenwald’s study, a WM region in the inferior part of the isthmus, probably belonging to the UF, presented bilateral significant correlations between DTI metrics (FA and MD) and tinnitus loudness. In our study, alterations of AFD in the structural white matter tracts were also elicited in the UF, mainly in the right hemisphere but also in the left UF mirroring the location in the right hemisphere, when considering a smaller segment extent. Although the group characteristics, the data acquisitions and the analysis pipelines differed between both studies, these concordant findings in the same locations might reflect a general change in the WM in this particular region. Throughout the literature, the UF appears to be involved with a number of neuropsychiatric disorders, including PTSD following TBI (Williamson et al., 2013), depression (Bracht et al., 2015, Taylor et al., 2007), social anxiety disorder (Bas-Hoogendam et al., 2016) and Alzheimer’s disease (Bozzali et al., 2016). Interesting here is the fact that the participants of the present study are free of neuropsychiatric disorders. We could thus hypothesize that the evolution of non-bothersome tinnitus of acoustic trauma origin toward tinnitus with anxiety co-morbidities might be mediated by the alteration of the uncinate fasciculus. We might also relate our findings to the alteration of the UF in PTSD following TBI (Williamson et al., 2013), since the tinnitus participants of the present study experienced another type of trauma, of acoustic origin. There might be a common neurological pathway of tinnitus of acoustic trauma origin, TBI and PTSD as suggested in a study of military population sample (Moring et al., 2018). Our results support the idea that the right and possibly left UF may be one of the substrates of this common pathway. Furthermore, as part of the posterior termination of the UF is located in the superior temporal gyrus (Hau et al., 2017), the increased AFD found in the tinnitus subjects here may reflect an increased connectivity with auditory areas, playing a direct role in the tinnitus perception.

The superior part of the isthmus between ventromedial prefrontal regions and temporal lobe, corresponding to the right IFO found in our study, could be related to a similar region from a DTI study in noise-induced tinnitus (Benson et al., 2014). Indeed, in this previous study, the authors found increased FA in the superior part of the isthmus, corresponding to right IFO. However, one must remain cautious when comparing with previous literature. Not only do the diffusion processing methods differ with our study, but there also is a nomenclature issue since the naming of the tracts has evolved with time in the literature, leading to increased difficulty in the comparison. The IFO is an associative tract, composed of long fibers that connect the frontal, the temporal, and the occipital lobes (Catani and Thiebautdeschotten, 2008, Conner et al., 2018, De Benedictis et al., 2014, Hau et al., 2016). This tract is thought to be a major contributor for language, attention and affective behavior but also to visual recognition system. Finally, IFO could be directly related to tinnitus percept through its direct connectivity to Heschl’s gyrus (Fernández et al., 2020).

Taken together, the implication of the isthmus of the UF and IFO in the right hemisphere supports the implication of the limbic system in chronic non-bothersome tinnitus subjects, confirming previous investigations.

### 4.3 Involvement of the superior parietal lobe

In our study the finding of increased AFD in the WM fanning underneath the superior parietal lobe is new in the tinnitus literature. The T-PAR is the parietal subdivision of the corona radiata that was found in the DTI study of noise-induced tinnitus (Benson et al., 2014) with an increased FA in tinnitus group in a location mentioned as left superior corona radiata, originating in Brodmann area 6 and terminating in the cortico-spinal tract (CST), thus potentially more anterior than what is found in our study. In the context of the tinnitus literature, other WM modifications in this region were not reported. The POPT is the tract connecting pontine nuclei with the parietal and occipital cortices, just posterior to the CST, which could also refer to Benson’s findings. Together with T-PAR, we found increased AFD in the fanning portions of these tracts in the left hemisphere, suggesting a possible involvement of superior parietal regions in the tinnitus perception after acoustic trauma. This brain area is known to be an associative cortical region since it combines inputs from a number of brain areas including somatosensory, auditory, visual, motor, cingulate and prefrontal cortices, and it integrates proprioceptive and vestibular signals from subcortical areas (Whitlock, 2017). The implication of superior parietal cortex found here supports the view of an auditory-somatosensory plasticity taking place in tinnitus (Wu et al., 2016).

### 4.4 Involvement of the acoustic radiations

From a methodological point of view, the tracking of auditory radiations remains a challenge (Maffei et al., 2019). In this study, a specific pipeline (Xtract) enabled us to investigate the acoustic radiations between medial geniculate nuclei and the auditory cortices. The involvement of auditory cortices in the tinnitus percept remains debated. A review of previous literature using the tensor model showed that the auditory cortex and inferior colliculus of tinnitus patients were found to display changes in diffusion metrics, such as fractional anisotropy, compared to normal hearing controls (Tarabichi et al., 2018). Furthermore, a higher FA and lower MD in anatomically defined regions of interest in the white matter tracts underneath both auditory cortices in tinnitus patients compared with control subjects was observed (Seydell-Greenwald et al., 2014). However, when taking into account confounding factors it was shown that the general fractional anisotropy in the primary auditory cortex was rather related to age, duration of tinnitus and hearing loss (Yoo et al., 2016). In our study, in line with Yoo and also with Husain (Husain et al., 2011), after regression for hearing loss, THI, and tinnitus duration, we did not find changes underneath the auditory cortex. However, we found a remaining decreased FA in right AR in tinnitus group located above the posterior ventricle in the bending part of the radiation (Fig. 4, Supp. Mat. S2). With AFD, we only found a weak difference below statistical significance at this same location (Supp. Mat. S1), somehow in coherence with the FA result.

### 4.5 Impact of cofactors

The literature reports WM tracts modifications with hearing loss in tinnitus subjects (Husain et al., 2011, Yoo et al., 2016). Yoo and collaborators found effects of age and hearing loss in widespread nonspecific regions. In this study, where age was not different between the two groups, we found that AFD was negatively impacted and decreased with hearing loss in two fiber bundles, the left ILF and the left FPT (Fig. 5), a result also observed with FA (Fig. 6). These correlations were found in the inferior pole of the left temporal lobe, while Husain and collaborators found a correlation with FA in the ILF of the right hemisphere at a location further up in the brain close to the posterior thalamic radiation. We also found significant correlation with hearing loss in the fanning part of fronto-pontine tract underneath the left dorsal frontal lobe, a result that was not found up to now in the literature. We did not find any correlation with hearing loss in the acoustic radiations. Looking for other influences of THI or tinnitus duration on AFD, we found no further significant correlation, in line with other studies (Schmidt et al., 2018).

Laterality was evoked as a potential explanation for differences in results between studies using very similar voxel based morphometry methodologies (Landgrebe et al., 2009; Mühlau et al., 2006). But Landgrebe’s study had unequal numbers of lateralized tinnitus, which would thus unlikely even out by averaging. In the present study with 13 bilateral tinnitus, 3 left sided tinnitus and 3 right sided tinnitus participants, we hypothesized that laterality would be compensated in the averaging of the group statistical analysis. Furthermore, the subgroup analysis on bilateral tinnitus led to results that confirm those of the whole group study. The results of the main analysis are therefore likely not driven by tinnitus laterality.

### 4.6 Metric comparison derived from single- and multiple-fibers diffusion models

The main metric of interest in this study was the integral of the FOD lobes, also referred to as AFD. This measure relates in each voxel to the total fiber density of a given fiber bundle. The FOD peak amplitude metric relates to the fiber density per unit solid angle in the direction closest to that determined by the Track Orientation Maps. Both measures can be interpreted in relationship with the intra-axonal volume, and this interpretability is improved at high b-values (Raffelt et al., 2017). Although the b-value used in the present study is typical for clinical explorations (1000 m/s^2^), this should figure among the criteria to take into account for future study design. Furthermore, the differences observed between these two metrics likely relate to fiber orientation dispersion.

As the previous literature on tinnitus related to white matter modifications mainly reported tensor derived metrics (mostly FA but also mean, axial and radial diffusivities). The profiles of FA values were also derived in our study for comparison with AFD and FOD peak amplitude and displayed (Fig. 7) for the control group. These FA values were mapped on the bundle specific tractography that deals with the issue of crossing fibers. In general, anisotropy related metrics are biased by the degree of coherence of fiber tract directions (Basser and Pierpaoli, 1996). This means that even if the segmentation is performed successfully, one may not be able to disentangle between real biological changes and amounts of crossing fibers or partial volume effects (Dell’Acqua and Tournier, 2019). The interpretation of tensor derived metrics in voxels where fibers cross therefore remains subject to caution. Despite these limitations, the profiles of AFD and FA along the FOIs were very close in many bundles, in particular in the right UF and in the right IFO. In the isthmus of the right UF and the right IFO, one main antero-posterior fiber direction exists that may explain these similarities. This may also explain why the difference between tinnitus subjects and controls were congruent with findings of previous authors using FA (Seydell-Greenwald et al., 2014). In a few bundles (bilateral AF, bilateral CG, bilateral POPT), the differences are located close to the origin or termination of the bundle where not only large bundles, but also small U-fibers are present. In others (bilateral AR, bilateral FPT), they are located along the course of the bundle. This is particularly the case with acoustic radiations in the region where they present a large bending, possibly explaining the differences in results with the literature where angle thresholds could be used.

## 5 Methodological strengths and limitations

The two main strengths of this study are its analysis of tinnitus perception *per se* and its state-of-the-art methodology for white matter connectivity analysis. The population of the tinnitus group present the same etiology with trauma induced tinnitus, non-bothersome tinnitus characteristics reflected by small THI values and excluding co-morbidities and known tinnitus duration providing a homogeneity in this group. The laterality of tinnitus doesn’t impact the results of the present study. The control group was matched in age and gender. They differ in hearing loss, and this factor was taken into account in the analysis. They also differ in their socio-professional backgrounds, with nine professional military tinnitus participants and no military in the controls. However, the military included in the study did not present any mTBI or PTSD or other psychiatric disorders, since these were exclusion criteria. They are therefore not comparable with the participants found in the literature on blast exposure in the military. This is an attempt to answer the request in the field of tinnitus research for more homogeneous groups of participants by design and confound mitigation (Bauer, 2018, Schmidt et al., 2018) and to further disentangle tinnitus perception from confounding factors that may explain the discrepancies in the results reported in the literature. The second strength relate to the methodology used here. Indeed, we acquired the diffusion data with high angular resolution (60 directions) in order to be able to better model the fiber orientation per voxel, this to better reflects the white matter organization and to be able to derive related metrics, that are AFD and FOD peak amplitudes. In addition, we used state of the art deep-learning approaches trained on high quality data from the HCP to segregate the different bundles of interest. On account of this approach, we think that the results of our study reflect tinnitus percept *per se* rather than its co-morbidities.

The comparison of our results with the literature remains a difficulty. Indeed, not only are the models in previous literature based on the hypothesis of a single bundle per voxel, but the statistical analysis often relies on the skeleton of white matter that may be merging different co-linear bundles. The nomenclature of the bundles is an additional issue since it is not standardized yet and a single region in a given bundle may have received different names throughout the literature. In this study, we used the nomenclature from Wasserthal and collaborators that is the most accurate to our knowledge nowadays (Wasserthal et al., 2018). Furthermore, the statistical results reported in the literature mainly present p-values but not the effect size or the power, thus limiting the confidence one can have in those results. Statistical power analysis and effect sizes with both AFD and amplitude argues in favor of robustness of the results presented in this study. Furthermore, the fornix and anterior commissure are important fiber bundles of the limbic system and could not be analyzed here. This is another shortcoming of the present study which needs to be addressed in future work.

The reproducibility of TractSeg’s deep learning based approach we used here for fiber tracking was tested along with other approaches during the TraCED challenge of the ISMRM diffusion study group (Nath et al., 2020). It performed outstandingly with respect to overlap measures (Dice scores > 0.8 for right UF and for right IFO) and reproducibility (ICC > 0.85 for right UF and > 0.8 for right IFO) which argues in favor of excellent robustness for this method and these bundles. The method was also shown to minimize angular error and maximize Dice scores for all 72 bundles when compared to other tract delineation and segmentation algorithms (Wasserthal et al., 2019). The fact that the anterior commissure and the fornix were not analyzed here constitutes a possible shortcoming of the present study. They are two bundles of importance in the limbic system which need to be addressed in the future. In addition, despite the state of the art methodology used, the AR reconstruction remains challenging and highly variable across subjects, even when taking a probabilistic tractography algorithm that can reconstruct the AR at b-values lower than 3000 s/mm^2^ (Maffei et al., 2019). Although the results we obtained in the AR did not allow us to firmly conclude on their involvement in tinnitus perception (p-value slightly above the 0.05 probabilistic threshold after correction for multiple comparisons, with a power above 0.5), the segmentation and tracking were successful in all participants which is encouraging for future work with higher resolution acquisitions.

In conclusion, we provide evidence of white matter changes in the limbic system involving the orbitofrontal cortex but not between structures previously reported in tinnitus literature (hippocampus, parahippocampus, amygdala), since no changes were found in the cingulum or in the anterior thalamic radiations. The fornix and anterior commissure could not be investigated with the present acquisition conditions, but this could be solved with higher resolution acquisitions, multi-shell and higher b values combined with the advanced methods used here. Furthermore, the exact plasticity mechanisms cannot be settled using the present cross sectional study paradigm and remains an open question for future research. Causal relationships between acoustic trauma, tinnitus, and white matter plasticity could be addressed using a longitudinal analysis.

## Author contributions

Experiment conception and design were carried out by A.J. and C.D.-M. Experiment execution was done by A.J. and C.D.-M. Data analysis was performed by C.J., A.A. and C.D.-M. Discussion of the study was carried out by A.A., A.J., C.J., and C.D.-M. Article writing was carried out by C.J., C.D.-M., and final version approved by all authors.

## Declaration of Competing Interest

The authors declare that they have no known competing financial interests or personal relationships that could have appeared to influence the work reported in this paper.
