# Supplementary material for: Tracking white-matter brain modifications in chronic non-bothersome acoustic trauma tinnitus
Source: Neuroimage Clin. 2021 May 8;31:102696. doi: 10.1016/j.nicl.2021.102696 (PMC8163994; doi:10.1016/j.nicl.2021.102696)
Supplement: Supplementary file 1 [file mmc1.zip › SuppMat_Tables_Artwort.odt]

Tracking white matter brain modifications in chronic non-bothersome acoustic trauma tinnitus

Chloé Jaroszynski1, Arnaud Attyé2, Agnès Job1,3, Chantal Delon-Martin1, *

1. Univ. Grenoble Alpes, Inserm, U1216, Grenoble Institut Neurosciences, 38000 Grenoble, France

2. Univ. Grenoble Alpes, CHU Grenoble Alpes, 38000 Grenoble, France

3. Institut de Recherche Biomédicale des Armées, IRBA, Brétigny s/Orge, France

* Corresponding author: Chantal Delon-Martin, [chantal.delon@univ-grenoble-alpes.fr](mailto:chantal.delon@univ-grenoble-alpes.fr)

Permanent address: Grenoble Institute of Neurosciences (GIN), INSERM U1216, Bâtiment Edmond J. Safra, Chemin F. Ferrini, Fr-38700 La Tronche – France

Supplementary Materials

Supp. Mat. Table 1 : Differences in AFD values between tinnitus group and control group along the 27 tracts of interest. Lines with light grey background correspond to bundles presenting at least 2 segments with significant difference between both groups.

| AFD |  |  |  |  |  |
| --- | --- | --- | --- | --- | --- |
|  |  |  |  |  |  |
| Bundle names | min_pvalue | Segment number | T-value | Cohen’s d | power |
|  |  |  |  |  |  |
| AR_right | 4.70E-05 | 29 | 4.623 | 1.541 | 0.554 |
| AR_left | 0.00037 | 22 | -3.930 | 1.310 | 0.312 |
| AF_right | 3.04E-06 | 57 | 5.520 | 1.840 | 0.827 |
| AF_left | 1.13E-07 | 11 | -6.593 | 2.198 | 0.972 |
| ATR_right | 1.20E-05 | 13 | 5.061 | 1.687 | 0.702 |
| ATR_left | 1.40E-05 | 55 | 5.023 | 1.674 | 0.690 |
| CC_6 | 2.97E-07 | 96 | -6.276 | 2.092 | 0.948 |
| CG_right | 4.08E-06 | 65 | -5.425 | 1.808 | 0.804 |
| CG_left | 9.28E-07 | 12 | -5.906 | 1.969 | 0.901 |
| FPT_left | 5.67E-07 | 12 | -6.066 | 2.022 | 0.924 |
| FPT_right | 0.000179 | 75 | 4.178 | 1.393 | 0.395 |
| IFO_right | 6.70E-08 | 17 | -6.764 | 2.255 | 0.980 |
| IFO_left | 6.65E-07 | 38 | -6.014 | 2.005 | 0.917 |
| ILF_left | 2.10E-05 | 15 | -4.897 | 1.632 | 0.649 |
| ILF_right | 0.00076 | 77 | 3.679 | 1.226 | 0.236 |
| OR_right | 1.90E-05 | 63 | 4.929 | 1.643 | 0.659 |
| OR_left | 0.000105 | 14 | -4.357 | 1.452 | 0.458 |
| POPT_left | 8.32E-07 | 10 | -5.942 | 1.981 | 0.907 |
| POPT_right | 9.70E-05 | 44 | 4.382 | 1.461 | 0.467 |
| STR_left | 4.30E-05 | 11 | -4.658 | 1.553 | 0.566 |
| STR_right | 0.044831 | 90 | -2.079 | 0.693 | 0.015 |
| UF_left | 3.43E-06 | 74 | -5.482 | 1.827 | 0.818 |
| UF_right | 4.95E-09 | 62 | -7.632 | 2.544 | 0.998 |
| T_PAR_left | 1.63E-08 | 11 | -7.233 | 2.411 | 0.993 |
| T_PAR_right | 8.20E-05 | 10 | -4.439 | 1.480 | 0.488 |
| T_OCC_left | 1.60E-05 | 51 | 4.985 | 1.662 | 0.678 |
| T_OCC_right | 0.0001 | 63 | 4.372 | 1.457 | 0.464 |

Supp. Mat. Table 2 : Differences in amplitudes values between tinnitus group and control group along the 27 tracts of interest. Lines with light grey background correspond to bundles presenting at least 2 segments with significant difference between both groups.

| Amplitudes |  |  |  |  |  |
| --- | --- | --- | --- | --- | --- |
|  |  |  |  |  |  |
| Bundle names | min p_value | Segment number | T-value | Cohen’s d | power |
|  |  |  |  |  |  |
| AR_right | 9.04E-06 | 24 | -5.166 | 1.722 | 0.740 |
| AR_left | 4.9E-08 | 85 | -6.868 | 2.289 | 0.985 |
| AF_right | 8.79E-06 | 56 | 5.175 | 1.725 | 0.743 |
| AF_left | 1.95E-06 | 33 | 5.665 | 1.888 | 0.863 |
| ATR_right | 4.48E-06 | 14 | 5.394 | 1.798 | 0.802 |
| ATR_left | 0.000132 | 58 | 4.281 | 1.427 | 0.438 |
| CC_6 | 5.85E-06 | 96 | -5.308 | 1.769 | 0.780 |
| CG_right | 0.000151 | 67 | -4.235 | 1.412 | 0.422 |
| CG_left | 6.62E-07 | 74 | -6.016 | 2.005 | 0.920 |
| FPT_left | 0.000218 | 12 | -4.111 | 1.370 | 0.379 |
| FPT_right | 0.000143 | 77 | 4.254 | 1.418 | 0.429 |
| IFO_right | 1.6E-09 | 17 | -8.017 | 2.672 | 0.999 |
| IFO_left | 1.5E-05 | 36 | -5.005 | 1.668 | 0.691 |
| ILF_left | 9.7E-05 | 15 | -4.382 | 1.461 | 0.475 |
| ILF_right | 2.7E-05 | 55 | 4.812 | 1.604 | 0.627 |
| OR_right | 0.001364 | 70 | 3.471 | 1.157 | 0.187 |
| OR_left | 0.000202 | 45 | 4.136 | 1.379 | 0.387 |
| POPT_left | 2.63E-06 | 9 | -5.568 | 1.856 | 0.843 |
| POPT_right | 6.5E-05 | 41 | 4.518 | 1.506 | 0.524 |
| STR_left | 8.13E-06 | 14 | -5.200 | 1.733 | 0.750 |
| STR_right | 0.02974 | 23 | -2.263 | 0.754 | 0.023 |
| UF_left | 7.87E-06 | 74 | -5.211 | 1.737 | 0.753 |
| UF_right | 8.02E-08 | 57 | -6.705 | 2.235 | 0.979 |
| T_PAR_left | 2.23E-07 | 9 | -6.371 | 2.124 | 0.958 |
| T_PAR_right | 0.000319 | 69 | 3.980 | 1.327 | 0.335 |
| T_OCC_left | 3.1E-05 | 50 | 4.761 | 1.587 | 0.610 |
| T_OCC_right | 0.000754 | 51 | 3.682 | 1.227 | 0.243 |

Supp. Mat. Table 3 : Differences in FA values between tinnitus group and control group along the 27 tracts of interest. Lines with light grey background correspond to bundles presenting at least 2 segments with significant difference between both groups.

| FA |  |  |  |  |  |
| --- | --- | --- | --- | --- | --- |
|  |  |  |  |  |  |
| Bundle names | min_pvalue | Segment number | T-value | Cohen’s d | power |
|  |  |  |  |  |  |
| AR_right | 1.30E-05 | 34 | 5.037 | 1.679 | 0.702 |
| AR_left | 1.55E-03 | 9 | 3.424 | 1.141 | 0.177 |
| AF_right | 2.40E-05 | 11 | 4.846 | 1.615 | 0.640 |
| AF_left | 2.83E-07 | 33 | 6.293 | 2.098 | 0.952 |
| ATR_right | 8.00E-05 | 91 | 4.447 | 1.482 | 0.499 |
| ATR_left | 1.20E-05 | 0 | -5.087 | 1.696 | 0.718 |
| CC_6 | 6.14E-06 | 96 | -5.292 | 1.764 | 0.777 |
| CG_right | 9.14E-06 | 65 | -5.162 | 1.721 | 0.740 |
| CG_left | 1.80E-05 | 87 | -4.933 | 1.644 | 0.669 |
| FPT_left | 3.45E-06 | 12 | -5.479 | 1.826 | 0.824 |
| FPT_right | 3.20E-05 | 75 | 4.749 | 1.583 | 0.607 |
| IFO_right | 1.64E-08 | 17 | -7.231 | 2.410 | 0.994 |
| IFO_left | 3.72E-04 | 36 | -3.928 | 1.309 | 0.319 |
| ILF_left | 8.11E-04 | 14 | -3.656 | 1.219 | 0.237 |
| ILF_right | 2.93E-04 | 80 | 4.009 | 1.336 | 0.346 |
| OR_right | 2.25E-04 | 66 | 4.100 | 1.367 | 0.377 |
| OR_left | 3.10E-08 | 51 | 7.018 | 2.339 | 0.990 |
| POPT_left | 2.22E-06 | 10 | -5.622 | 1.874 | 0.855 |
| POPT_right | 2.53E-04 | 46 | 4.060 | 1.353 | 0.363 |
| STR_left | 9.53E-06 | 13 | -5.149 | 1.716 | 0.736 |
| STR_right | 4.51E-03 | 10 | -3.029 | 1.010 | 0.099 |
| UF_left | 4.70E-05 | 75 | -4.628 | 1.543 | 0.565 |
| UF_right | 1.86E-06 | 62 | -5.680 | 1.893 | 0.867 |
| T_PAR_left | 6.50E-08 | 9 | -6.775 | 2.258 | 0.982 |
| T_PAR_right | 5.70E-05 | 69 | 4.562 | 1.521 | 0.541 |
| T_OCC_left | 2.44E-08 | 50 | 7.099 | 2.366 | 0.991 |
| T_OCC_right | 5.43E-04 | 33 | 3.797 | 1.266 | 0.278 |

Supp. Mat. S1 

AFD profiles and analysis results with 2 consecutive segments as threshold after correction for multiple corrections. The red dotted line indicates the location where the pvalue of the t-test was below the alphaFWE threshold for at least 2 consecutive segments.   

Supp. Mat. S2 

Anatomy and segmentation of the Acoustic Radiation in an example participant. The region were the group statistic elicited an FA decrease in tinnitus participants is represented by the red overlay. The same tendancy was observed for AFD, although it did not reach the statistical threshold.

Supp. Mat. S3

FOD_peak_amplitude profiles of all fiber bundles included in the analysis. The red dotted line indicates the location of significant differences between the profiles pour tinnitus and controls, with a minimum of 5 consecutive segments.

Supp. Mat. S4

FA profiles of all the fiber bundles in the analysis. The red dotted line indicates the location of significant differences between the profiles pour tinnitus and controls, with a minimum of 5 consecutive segments.
